# Characterization of the Conditioned Medium from Amniotic Membrane Cells: Prostaglandins as Key Effectors of Its Immunomodulatory Activity

**DOI:** 10.1371/journal.pone.0046956

**Published:** 2012-10-10

**Authors:** Daniele Rossi, Stefano Pianta, Marta Magatti, Peter Sedlmayr, Ornella Parolini

**Affiliations:** 1 Centro di Ricerca E. Menni, Fondazione Poliambulanza-Istituto Ospedaliero, Brescia, Italia; 2 Doctoral School of Molecular Medicine, University of Milan, Milano, Italia; 3 Institute of Cell Biology, Histology and Embryology, Center for Molecular Medicine, Medical University of Graz, Graz, Austria; University of Pittsburgh, United States of America

## Abstract

We previously demonstrated that cells isolated from the mesenchymal region of the human amniotic membrane (human amniotic mesenchymal tissue cells, hAMTC) possess immunoregulatory roles, such as inhibition of lymphocyte proliferation and cytokine production, and suppression of generation and maturation of monocyte-derived dendritic cells, as reported for MSC from other sources. The precise factors and mechanisms responsible for the immunoregulatory roles of hAMTC remain unknown. In this study, we aimed to identify the soluble factors released by hAMTC and responsible for the anti-proliferative effect on lymphocytes, and the mechanisms underlying their actions, *in vitro*. Conditioned medium (CM) was prepared under routine culture conditions from hAMTC (CM-hAMTC) and also from fragments of the whole human amniotic membrane (CM-hAM). We analyzed the thermostability, chemical nature, and the molecular weight of the factors likely responsible for the anti-proliferative effects. We also evaluated the participation of cytokines known to be involved in the immunomodulatory actions of MSC from other sources, and attempted to block different synthetic pathways. We demonstrate that the inhibitory factors are temperature-stable, have a small molecular weight, and are likely of a non-proteinaceous nature. Only inhibition of cyclooxygenase pathway partially reverted the anti-proliferative effect, suggesting prostaglandins as key effector molecules. Factors previously documented to take part in the inhibitory effects of MSCs from other sources (HGF, TGF-β, NO and IDO) were not involved. Furthermore, we prove for the first time that the anti-proliferative effect is intrinsic to the amniotic membrane and cells derived thereof, since it is manifested in the absence of stimulating culture conditions, as opposed to MSC derived from the bone marrow, which possess an anti-proliferative ability only when cultured in the presence of activating stimuli. Finally, we show that the amniotic membrane could be an interesting source of soluble factors, without referring to extensive cell preparation.

## Introduction

Adult mesenchymal stromal/stem cells (MSC), first identified in bone marrow (BM) [1], have also been successfully isolated from various other sources, including umbilical cord blood [2], adipose tissue [3], peripheral blood [4], amniotic fluid [5], Wharton’s Jelly [6], [7], and more recently from different tissue of human placenta [8].

MSC have been extensively studied both for their ability to differentiate towards multiple lineages, such as cells of the bone, cartilage, muscle, adipose, tendons and central nervous system [9]–[11], and for their interesting immunoregulatory properties [12]–[14]. Indeed, numerous *in vitro* studies have demonstrated that MSC can target and modulate the function of different cells of the immune system, such as T cells [15], B cells [16], natural killer cells [17] and dendritic cells [18], [19].

These *in vitro* immunomodulatory properties have generated enormous interest in the potential application of MSC *in vivo* as an immunosuppressive cellular therapy. Successful results have been obtained with the use of MSC for both the prevention of graft-versus-host disease (GVHD) in solid organ transplantation [20], [21] and for the treatment of steroid-resistant acute GVHD, arising after allogeneic hematopoietic cell transplantation [22], [23]. Moreover, systemic infusion of MSC ameliorated the clinical and histopathological severity of experimental autoimmune encephalomyelitis [24], multiple sclerosis [25] and colitis [26].

**Figure 1:**
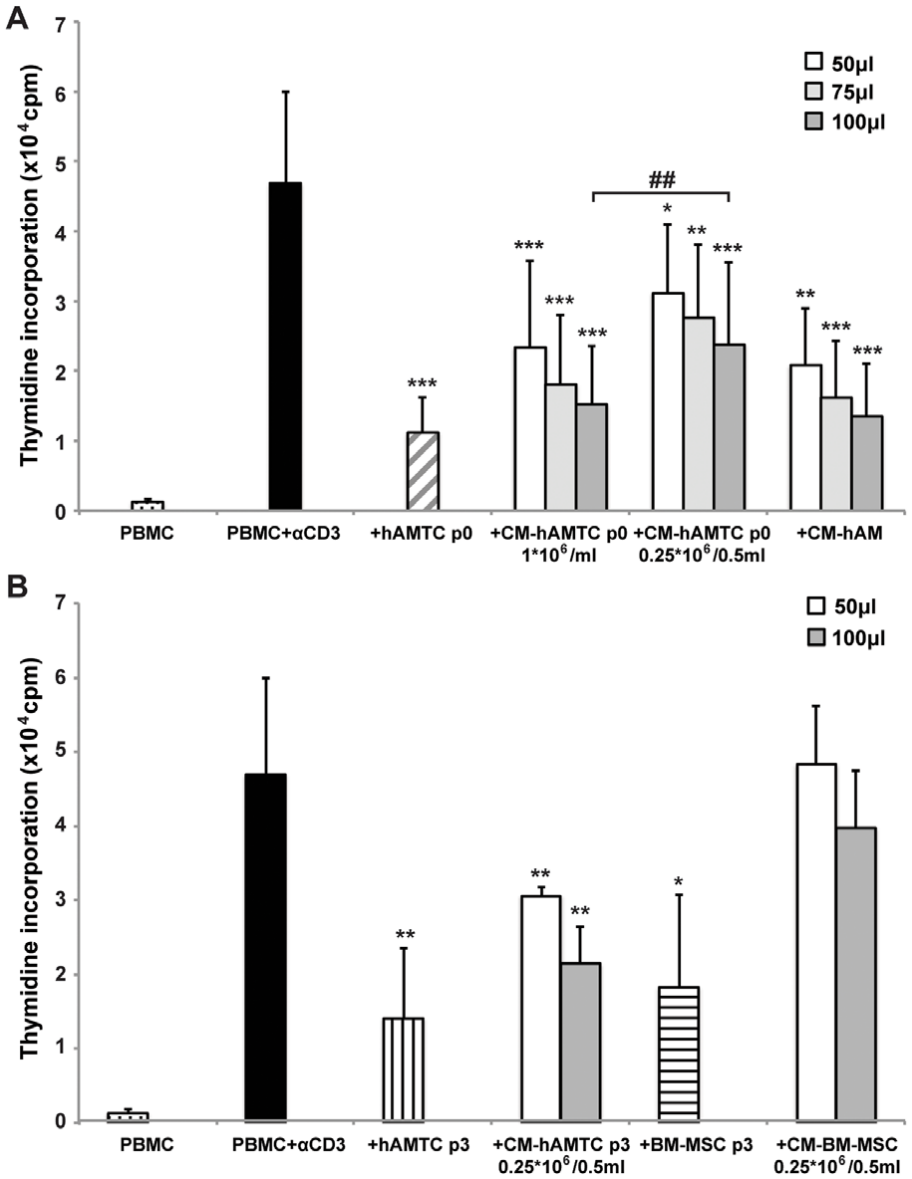
Analysis of inhibitory effect of hAMTC, BM-MSC and of the respective CMs. Effects of the following on the proliferation of anti-CD3-stimulated lymphocytes (PBMC + αCD3): (**A**) hAMTC at passage 0 (white-grey striped bar), different amounts (50–75–100 μl) of CM obtained from the culture of hAMTC at p0 when plated at density of 1×10^6^ cells/ml (CM-hAMTC p0 1*×10^6^/ml) or 0.25×10^6^ cells/0.5 ml (CM-hAMTC p0 0.25*×10^6^/0.5 ml) and different amounts (50–75–100 μl) of CM-hAM; (**B**) hAMTC at passage 3 (vertical black line bar), BM-MSC at passage 3 (horizontal black line bar) and different amounts (50–100 μl) of CM derived from the culture of these cells. Data represent the mean and standard deviation (SD) of at least four independent experiments. * = p<0.05, ** = p<0.01, *** = p<0.001 versus PBMC + αCD3; ## = p<0.01.

Despite the fact that both *in vitro* and *in vivo* studies have provided support for the immunoregulatory role of MSC, the exact mechanisms whereby this immunomodulation is performed still remain to be fully elucidated. Both cell-to-cell contact and the secretion of bioactive soluble molecules seem critical for MSC’s regulatory action, but which modality is involved is still the object of a controversial debate [13], [14]. In addition, the mechanisms underlying MSC immunoregulatory actions seem to vary between species. For example, while soluble factors are essential for enhancing the suppressive effects of human MSC [15], [27], the immunomodulatory effect of rodent MSC seems to be prevalently mediated by cell-to-cell contact [28]. Furthermore, Ren and colleagues have reported that even soluble factor-mediated immunomodulation may depend on species-specific mechanisms [29].

Several factors are thought to participate in the induction of MSC-mediated immunosuppression, such as indoleamine 2,3-dioxygenase (IDO) enzyme, nitric oxide (NO), prostaglandin E2 (PGE2), tumor growth factor (TGF)-β, interleukin (IL)-10, hepatocyte growth factor (HGF) and galectins [13]. Nevertheless, conflicting results have been reported in regards to the specific contribution of these factors in the MSC-mediated regulatory process [13], [14].

**Figure 2:**
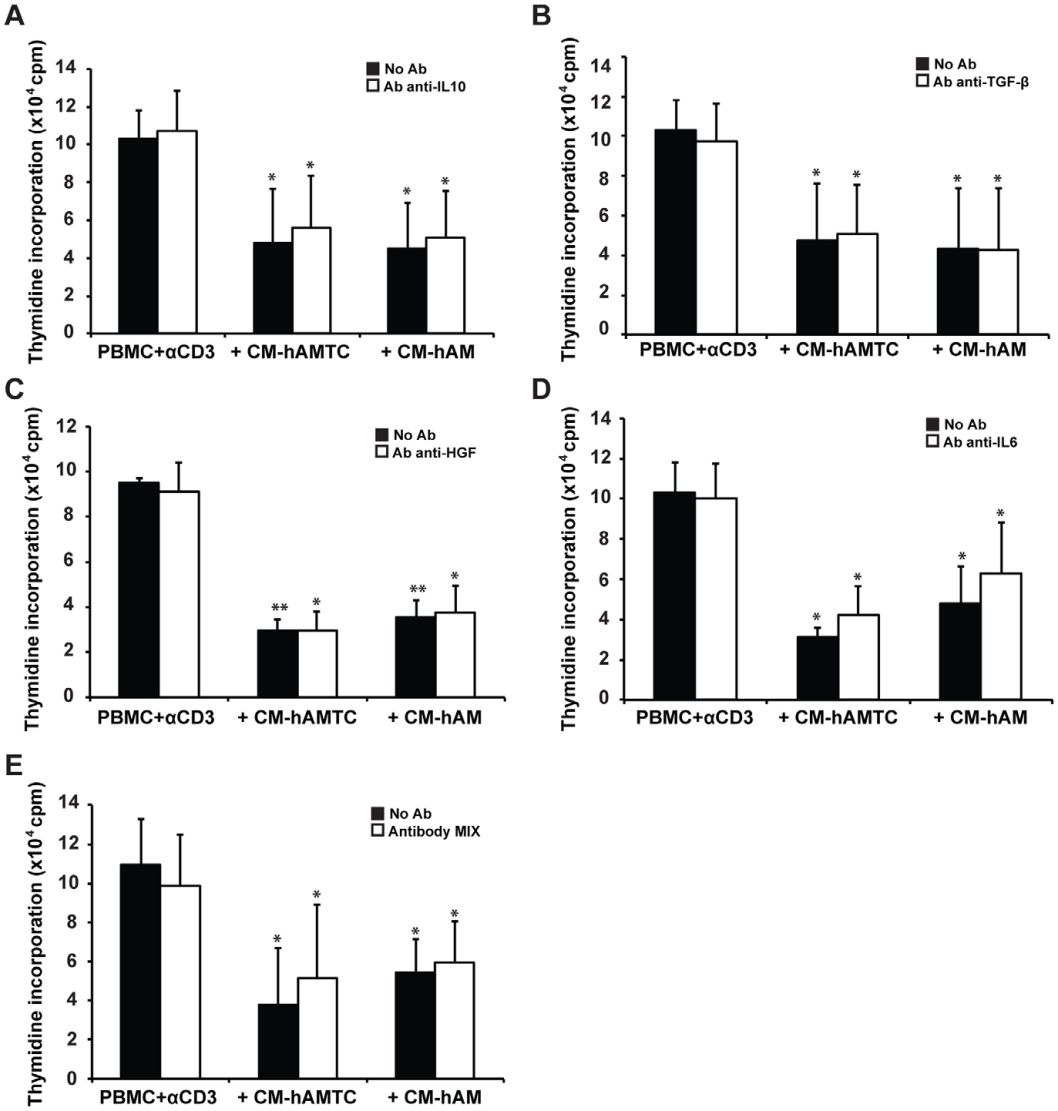
Inhibitory effect of CM-hAMTC and CM-hAM in presence of neutralizing antibodies. Lymphocyte proliferation, stimulated with anti-CD3 (PBMC + αCD3), in presence of 100 μl of control medium or CM with (white bars) or without (black bars) the addition of neutralizing antibodies against IL-10 (**A**), TGF-β (**B**), HGF (**C**), IL-6 (**D**), mix of antibodies anti-IL-10, -TGF-β, -HGF and -IL-6 (**E**). Data represent the mean and SD of at least four independent experiments. * = p<0.05, ** = p<0.01 versus PBMC + αCD3.

We have previously demonstrated that cells with characteristics of MSC can be successfully isolated from the human amniotic and chorionic membranes [30], [31]. In particular, we have shown through *in vitro* studies that cells isolated from the mesenchymal tissue of human amniotic membrane (herein referred to as hAMTC, for human amniotic mesenchymal tissue cells), inhibit lymphocyte proliferation [30], [32] and suppress the generation and maturation of monocyte-derived dendritic cells [18]. We have also shown that hAMTC abolish the production of inflammatory cytokines [18], likely through the action of yet unknown soluble inhibitory factor(s). In support of a key role of soluble factors in the suppressive action of amniotic membrane-derived cells, by using a mouse model of lung fibrosis we have shown that a reduction of severity and progression of the disease can be obtained both after the transplantation of amniotic membrane-derived cells and after the injection of their conditioned medium alone [33], [34]. Likewise, we have shown that the beneficial effects observed after the application of the amniotic membrane onto ischemic rat heart [35] and on fibrotic rat liver [36] are very likely induced by paracrine actions exerted on host tissues by bioactive molecules secreted by the amniotic cells, further suggesting the involvement of soluble factors as the key actor of the anti-inflammatory and anti-fibrotic effects of the amniotic cells.

In this study we set out to identify the immunomodulatory soluble factors released by hAMTC and the mechanisms underlying their actions, *in vitro*. To this aim, we prepared conditioned medium (CM) from the culture of hAMTC and fragments of the whole human amniotic membrane (hAM), and analyzed their immunomodulatory properties after different treatments, including temperature changes, proteinase treatment, fractionation and potential enrichment of immunosuppressive factor(s), and after inhibition of specific synthetic pathways.

**Figure 3:**
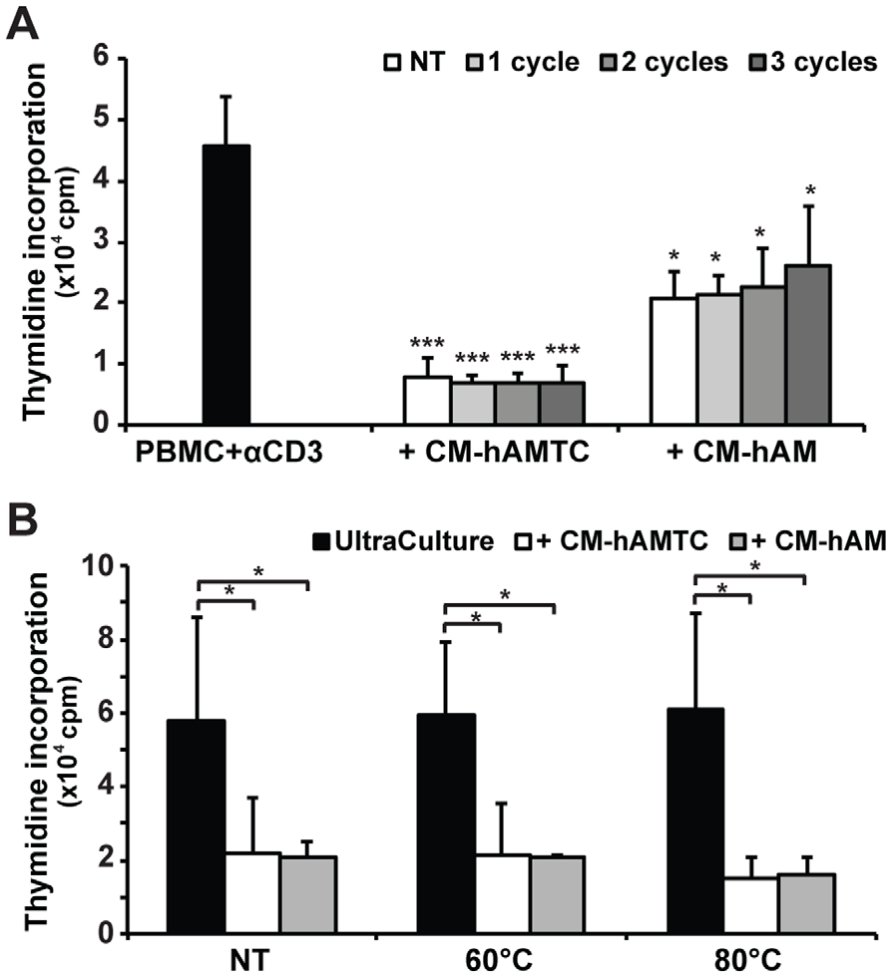
Effects of temperature changes on the anti-proliferative potential of CM-hAMTC and CM-hAM. Effects of the following on the proliferation of anti-CD3-stimulated lymphocytes (PBMC + αCD3): (**A**) 100 μl of untreated CM-hAMTC and CM-hAM (white bars) and of the corresponding CMs subjected to different frost-defrost cycles (grey bars); (**B**) 100 μl of untreated (NT) control medium (complete UltraCulture), CM-hAMTC, CM-hAM, and after incubation for 30 min at 60°C or 80°C. Data represent the mean and SD of at least three independent experiments. * = p<0.05, *** = p<0.001 versus PBMC + αCD3.

## Materials and Methods

### Ethics Statement

Human term placentas, bone marrow (BM) aspirates and peripheral blood were obtained from donors after informed consent according to the guidelines of Ethical Committee of the Catholic Hospital (CEIOC). For all the samples used throughout our study, informed written consent was sought and obtained from each single donor. The research project was authorized by Centro di Ricerca E. Menni-Fondazione Poliambulanza Commitee, which specifically approved this study.

**Figure 4:**
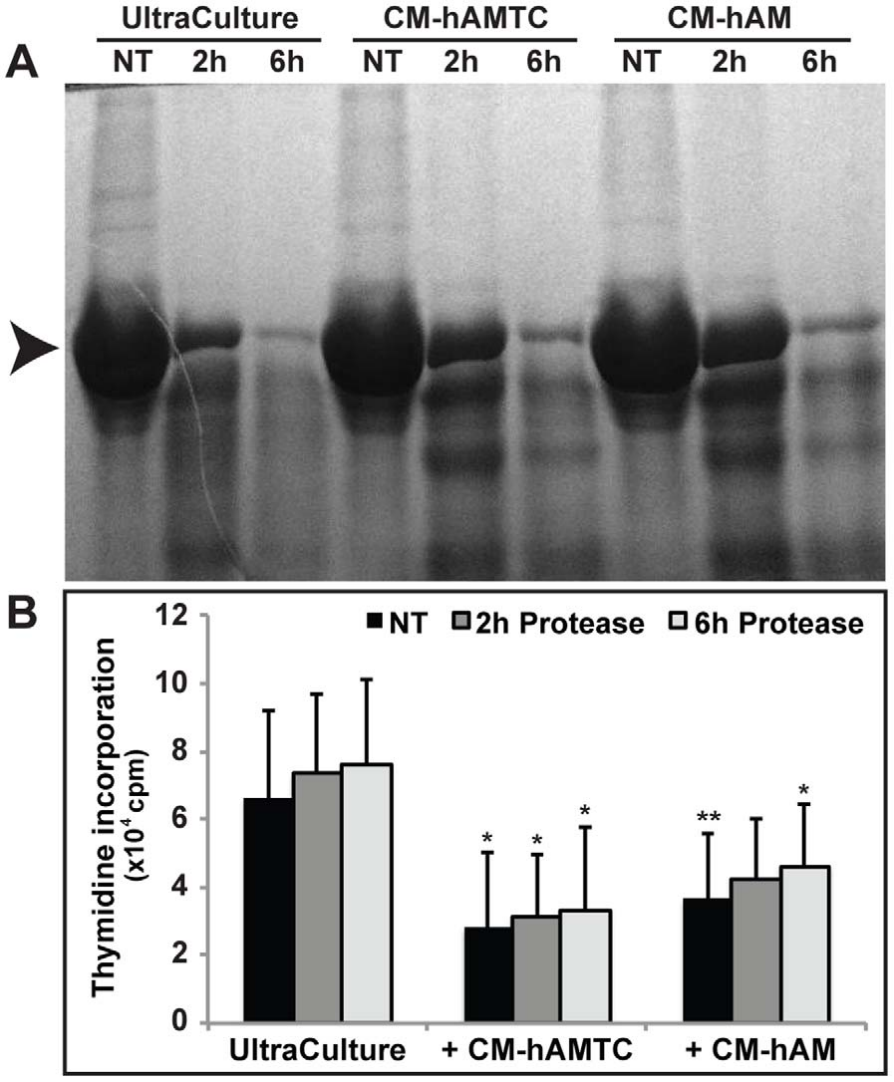
Effects of proteinase K treatment on the inhibitory activity of CM-hAMTC and CM-hAM. (**A**) Representative SDS-PAGE of untreated and proteinase K-treated (for 2 and 6 hrs) UltraCulture medium, CM-hAMTC and CM-hAM. Digestion level was calculated as the reduction of the density of the BSA band (arrow). (**B**) Inhibitory effect on lymphocyte proliferation, induced by anti-CD3 stimulation, of 100 μl of untreated (NT) and proteinase K-treated UltraCulture medium, CM-hAMTC and CM-hAM. Similar results were also obtained using 50 μl of CMs (data not shown). Data represent the mean and SD of at least five independent experiments. * = p<0.05, ** = p<0.01 versus UltraCulture.

### Human Sample Collection, Cell Isolation and Culture

#### Isolation and culture of human amniotic mesenchymal tissue cells (hAMTC)

Human term placentas were processed immediately after birth using a previously described protocol [31], with some modifications. The amnion was manually separated from the chorion and washed extensively in phosphate-buffered saline (PBS; Sigma, St Louis, MO, USA) containing 100 U/ml penicillin and 100 μg/ml streptomycin (herein referred to as P/S), (both from Euroclone, Whetherby, UK) and 2.5 μg/ml amphotericin B (Lonza, Basel, CH). Afterwards, the amnion was cut into small pieces (3×3 cm^2^). Amnion fragments were sterilized by a brief incubation in PBS +2.5% Eso Jod (Esoform, Italy) and 3 min in PBS containing 500 U/ml penicillin, 500 μg/ml streptomycin, 12.5 μg/ml amphotericin B and 1.87 mg/ml Cefamezin (Pfizer, Italy). Sterilized amnion fragments were then incubated for 9 min at 37°C in HBSS (Lonza, Basel, CH) containing 2.5 U/ml dispase (Roche, Mannheim, Germany). After a 3 min resting period at room temperature in RPMI 1640 medium (Cambrex, Verviers, Belgium) supplemented with 10% heat-inactivated fetal bovine serum (FBS; Sigma), 2 mM L-glutamine (Cambrex) and P/S, the fragments were digested with 0.94 mg/ml collagenase (Roche) and 20 μg/ml DNase (Roche) for 2.5–3 hrs at 37°C. Amnion fragments were then removed, mobilized cells were passed through a 100 μm cell strainer (BD Falcon, Bedford, MA), and collected by centrifugation at 200×g for 10 min. These cells, as reported above, are referred to as hAMTC, for human amniotic mesenchymal tissue cells. At passage 0 their phenotype is: CD90 (82%±3%), CD73 (66±6%), CD13 (89±2%), CD44 (57±10%), CD105 (6±4%), CD166 (14±4%), CD45 (6±3%), HLA-DR (6±3%), CD14 (6±3%) and negative to CD34 [37].

To obtain hAMTC at different passages, freshly isolated cells were plated at a density of 50×10^3^/cm^2^. Upon reaching confluency, adherent cells were trypsinized and then sub-cultured at a density of 20×10^3^/cm^2^ untill passages 3.

**Figure 5:**
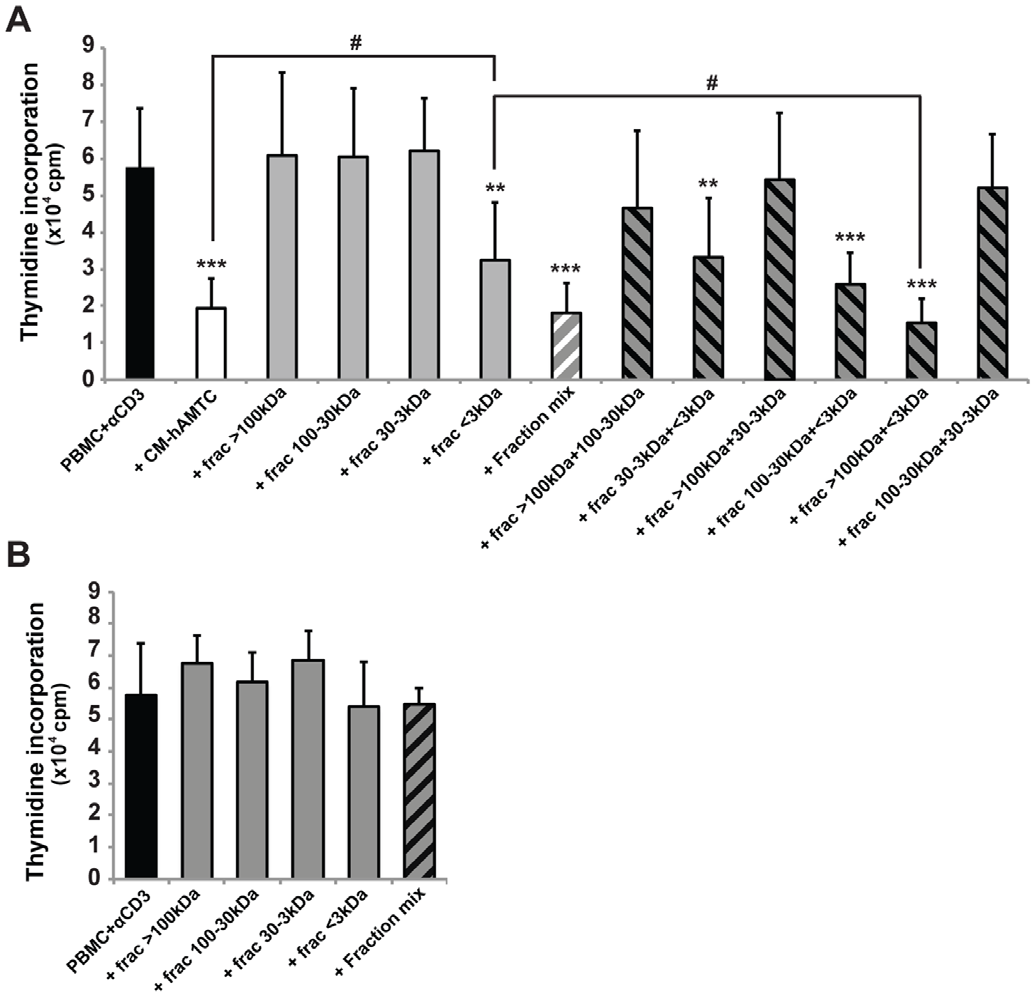
Study of the potential molecular weight of the inhibitory factor(s). (**A**) Lymphocyte proliferation, stimulated by anti-CD3, in presence of 100 μl of CM-hAMTC (white bar), CM-hAMTC-fraction-mix (grey bars) or mix of fractions with different molecular weights (white-grey- and black-grey-striped bars). (**B**) Lymphocyte proliferation, stimulated by anti-CD3, in presence of 100 μl of complete UltraCulture medium (black bar) or its fractions (grey and black-grey-striped bars). Data represent the mean and SD from at least six independent experiments. ** = p<0.01, *** = p<0.001 versus PBMC + αCD3; # = p<0.05.

#### BM-derived MSC isolation and culture

BM aspirates were extracted from the femoral heads of patients undergoing orthopedic surgery and processed as follows. Briefly, BM samples were diluted (1∶4) in PBS + P/S, transferred to 50 ml conical tubes (Falcon) and centrifuged at 1500×g for 15 min. After discarding of the fat layer and supernatant, the cells were layered on a density gradient (Lymphoprep, Axis Shield) and centrifuged at 670×g for 30 min. Recovered mononuclear cells were then collected, washed twice with PBS and centrifuged at 200×g for 10 min before being plated at a density of 8×10^5^/cm^2^ in DMEM (Lonza), supplemented with 10% heat-inactivated FBS, L-glutamine and P/S and maintained in culture at 37°C with 5% CO_2_. After 3 days, non-adherent cells were removed. Upon reaching confluency, adherent cells (BM-MSC) were trypsinized and sub-cultured at a density of 8×10^3^/cm^2^, and were used for experiments at passage 3.

**Figure 6:**
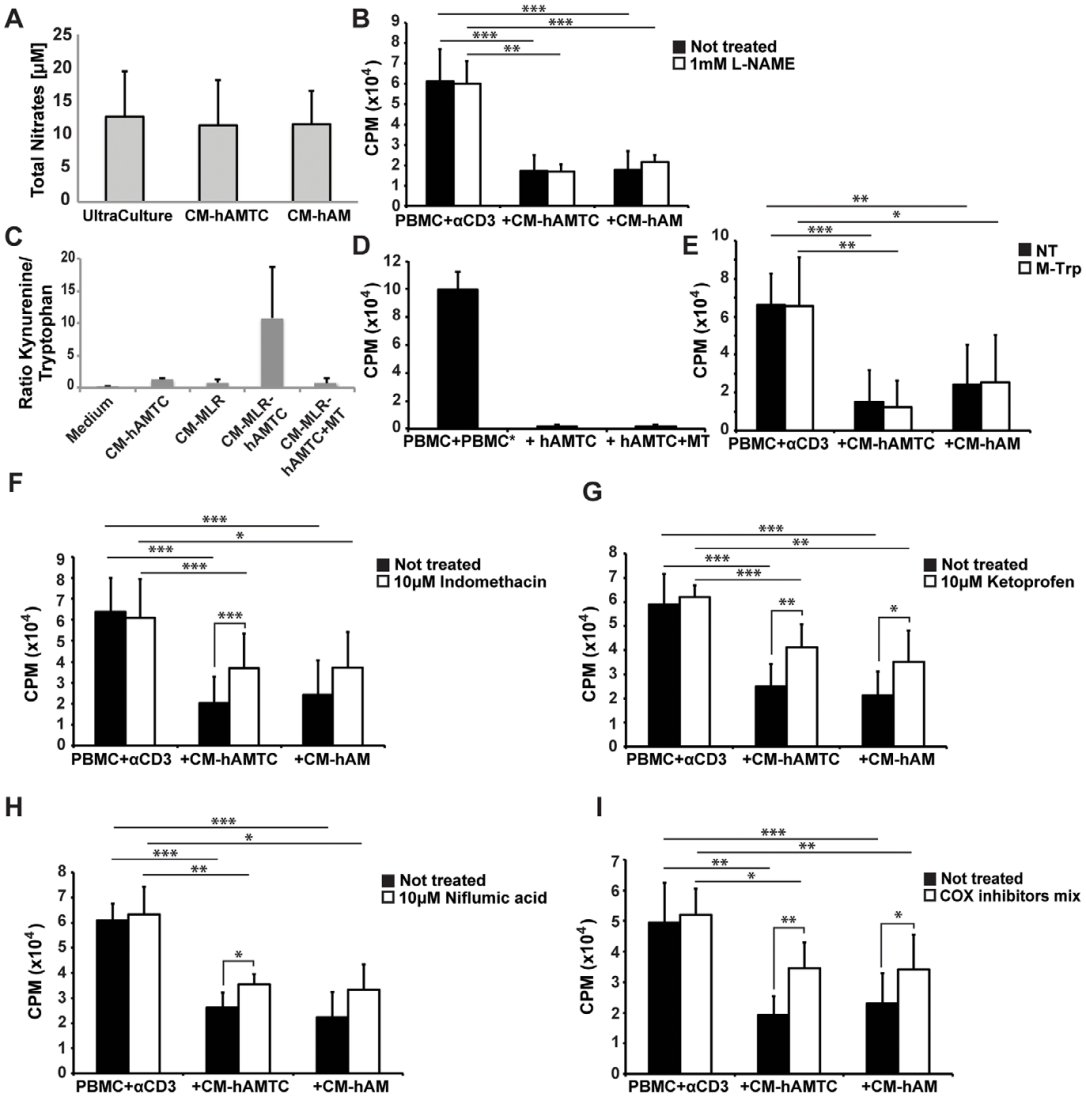
NO, kynurenine, and prostaglandins (PGs) in CM-hAMTC and CM-hAM. (**A**) NO quantification (total nitrate quantification) in UltraCulture, CM-hAMTC, and CM-hAM performed by means of the Griess test. (**B**) Lymphocyte proliferation (expressed in cpm) after stimulation with anti-CD3 and in presence of 100 μl of complete UltraCulture medium, CM derived from untreated (black bar), or L-NAME-treated (white bar) hAMTC and hAM cultures. (**C**) IDO activity, shown as the ratio between the concentration of kynurenine and tryptophan, in the control medium, CM-hAMTC, CM-MLR or CM-MLR-hAMTC, and in the presence of 0.5 mM DL-methyl-tryptophan (CM-MLR-hAMTC+MT). (**D**) Lymphocyte proliferation (expressed in cpm), induced by MLR, in the presence of hAMTC cultured with or without the addition of DL-methyl-tryptophan. (**E**) Lymphocyte proliferation (expressed in cpm), stimulated with anti-CD3, in presence of 100 μl of control medium or CM derived from untreated (black bar) or 0.5 mM DL-methyl-tryptophan-treated (white bar) hAMTC and hAM cultures. (**F–I**) Lymphocyte proliferation (expressed in cpm) after stimulation with anti-CD3, in presence of 100 μl of control medium or CM derived from untreated (black bar) or indomethacin- (**F**), ketoprofen- (**G**), niflumic acid- (**H**) or inhibitors-mix- (**I**) treated (white bars) hAMTC and hAM cultures. Data represent the mean and the SD of at least three independent experiments. * = p<0.05, ** = p<0.01, *** = p<0.001.

#### Human peripheral blood mononuclear cells (PBMC)

PBMC were obtained from heparinized whole blood samples or buffy coats from healthy subjects using density gradient centrifugation (Lymphoprep, Axis-Shield).

**Figure 7:**
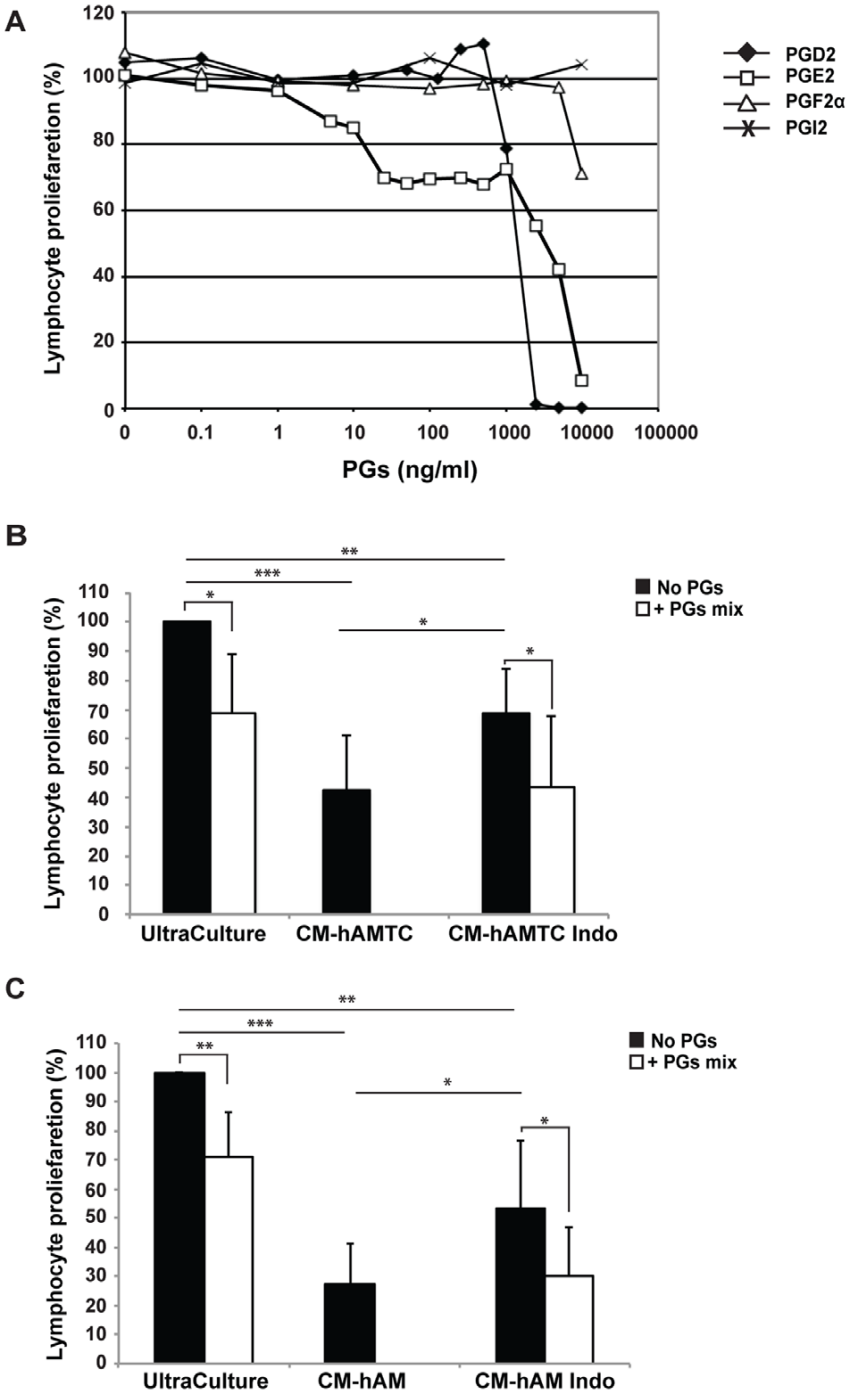
Effect of PGs on the proliferation of stimulated PBMC in presence or absence of CMs obtained from hAMTC or hAM treated or not with indometahcin. (**A**) Lymphocyte proliferation, stimulated with anti-CD3, in presence of different amount of prostaglandin (PG) PGD2 (⧫), PGE2 (□), PGF2α (**Δ**) or PGI2 (X). (**B**) Lymphocyte proliferation, stimulated with anti-CD3, in presence of 100 μl of control medium (UltraCulture) or CM derived from untreated (CM-hAMTC) or indomethacin treated (CM-hAMTC Indo) hAMTC with (white bars) or without (black bars) the addition of a mix of PGs (PGE2 500 ng/ml + PGD2 100 ng/ml + PGF2α 25 ng/ml). (**C**) Lymphocyte proliferation, stimulated with anti-CD3, in presence of 100 μl of control medium (UltraCulture) or CM derived from untreated (CM-hAM) or indomethacin treated (CM-hAM Indo) hAM with (white bars) or without (black bars) the addition of a mix of PGs (PGE2 25 ng/ml + PGD2 6 ng/ml + PGF2α 10 ng/ml). * = p<0.05, ** = p<0.01, *** = p<0.001.

### Production of Conditioned Medium (CM)

The different conditioned media (CMs) used in this study were obtained as follows.

*CM generated from freshly isolated hAMTC:* hAMTC (obtained from amniotic membranes of at least 80 different donors) at passage 0 were re-suspended in an opportune volume of complete UltraCulture medium, composed of UltraCULTURE Serum-free Medium (Lonza) supplemented with P/S, and plated in 24-well plates (Corning Inc., Corning, NY) at 2 different concentration: usually at 1×10^6^ cells/well in a final volume of 1 ml (referred to as CM-hAMTC) or, when otherwise specified, 2.5×10^5^ cells/well in a final volume of 0.5 ml (named CM-hAMTC p0 0.25*10^6^/0.5 ml).

*CM generated from hAMTC at passage 3 (CM-hAMTC p3):* hAMTC at p3 (obtained from 3 different placentas) were plated in 24-well plates at a density of 2.5×10^5^ cells/well in 0.5 ml of complete UltraCulture medium.

*CM generated from the culture of fragments of the amniotic membrane (CM-hAM):* sterilized fragments (3 cm×3 cm) from freshly separated amniotic membranes (obtained from at least 50 different donors) were plated (1 fragment in each well) in 6-wells plates (Corning Inc., Corning, NY) covered with a glass slide (Bio-Optica) and cultured in presence of 1 ml of complete UltraCulture medium. One fragment of amniotic membrane contains a number of hAMTC comparable to that used for the production of CM-hAMTC (8±4×10^5^ hAMTC/ml).

*CM generated from BM-MSC (CM-BM-MSC):* BM-MSC (obtained from 3 different BM donors) cultured untill passage 3, were plated in 24-well plates at a density of 2.5×10^5^ cells/well in 0.5 ml of complete UltraCulture medium.

*CM from cells that have been treated to block specific synthetic pathways:* hAMTC (at passage 0) and hAM fragments (both obtained from at least 8 different donors), were cultured in presence of the inhibitor of IDO activity (0.5 mM DL-methyl-tryptophan), the inhibitor of NO synthase (1 mM L-NAME) or the inhibitors of cyclooxygenase (indomethacin, ketoprofen, niflumic acid), all purchased from Sigma-Aldrich. To determine the optimal dose of COX inhibitors able to decrease the quantity of all prostanoids in both CM-hAMTC and CM-hAM, we previously performed experiments with different concentrations of the different COX inhibitors (*i.e.* 1, 5, 10, 50 and 100 μM), (data not shown). We found that the highest reversion of the inhibitory effect was obtained when using a concentration of 10 μM, which was the dose selected for further analysis.

All the cultures described above were performed at 37°C and 5% CO_2_ atmosphere, for 5 days. At the end of this culture period, the different CMs were collected, centrifuged at 300×g, filtered through a 0.8 μm sterile filter (Sartorius), opportunely aliquoted under sterile conditions and frozen at −80°C until use.

Each CM preparation was tested for its ability of inhibiting lymphocyte proliferation, as explained below in the paragraph describing lymphocyte proliferation test. For temperature stability analysis, proteinase K treatment and CM fractionation (described below), in order to obtain results that were less influenced by single donor variability and more representative of soluble factors released by hAMTC and hAM, we pooled 8 to 10 different CM-hAMTC or CM-hAM and used these pools for each specific analysis.

**Figure 8:**
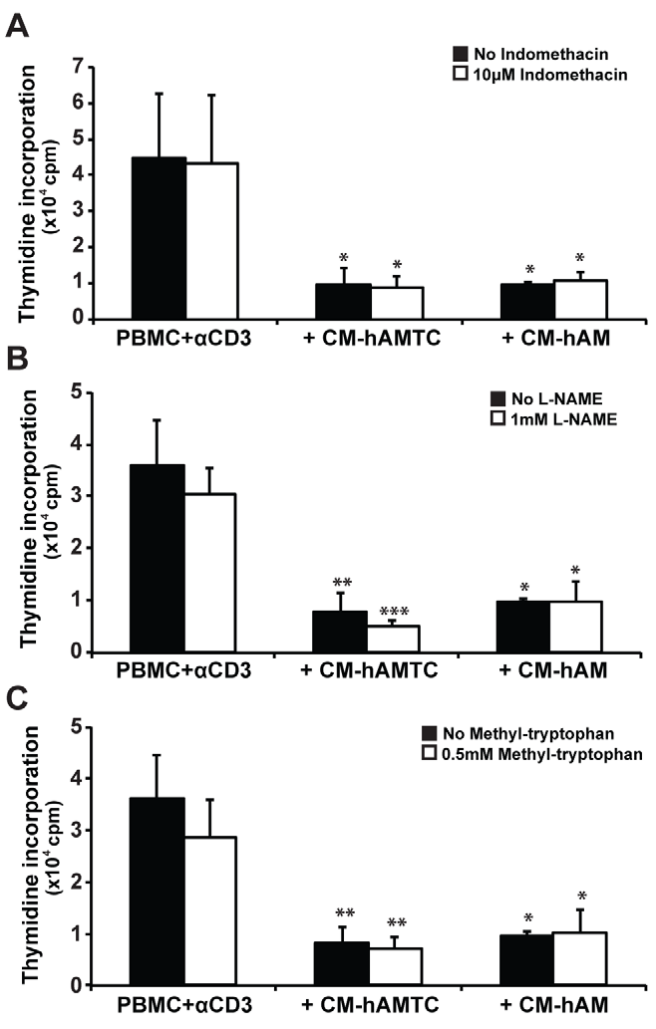
Inhibition of production of NO, kynurenine, and prostaglandins (PGs) in PBMC culture. Lymphocyte proliferation, stimulated with anti-CD3, in presence of 100 μl of control medium or CM-hAMTC and CM-hAM with (white bars) or without (black bars) the addition of indomethacin (**A**), L-NAME (**B**) or DL-methyl-tryptophan (**C**). Data represent the mean and SD of at least three independent experiments. * = p<0.05, ** = p<0.01, *** = p<0.001 versus PBMC + αCD3.

### Temperature Stability Analysis

CM-hAMTC and CM-hAM were subjected to different temperature changes. In particular, they were: *i)* incubated for 30 min at 60°C or 80°C; *ii)* subjected to three frost-defrost cycles, which were carried out by putting these CMs in liquid nitrogen and subsequently in a thermostatic bath at 37°C.

### Proteinase K Treatment

Complete UltraCulture medium, CM-hAMTC and CM-hAM (1 ml of each sample) were treated with 1 U of proteinase K linked to agarose beads (Sigma-Aldrich), for 2 and 6 hours at 37°C. The digestion was then blocked by removing proteinase K through brief centrifugation. Proteinase-treated CMs were loaded on SDS-PAGE gels that were then stained by Coomassie blue. The efficacy of protein digestion was evaluated monitoring the reduction of the density of the band corresponding to BSA protein, which is the most abundant in CMs and complete UltraCulture medium. The band intensity was quantified using the Quantity One software (Bio-Rad).

### CM Fractionation

To obtain fractions potentially enriched in soluble factor(s) with inhibitory effects, different CM-hAMTC (n = 11 pools) were fractionated using Centrifugal Filter Devices with different NMWL (Nominal Molecular Weight Limit) Ultracel membranes (Millipore). First, 3 ml of CM-hAMTC were loaded onto a Centrifugal Filter Device with Ultracel membranes with a 100 kDa cut-off and centrifuged at 2700×g for 35 min. Then, the solute was re-suspended in the same volume of complete UltraCulture medium as the loaded sample, while the eluate was loaded onto a column with a 30 kDa membrane cut-off. After centrifugation at 2700×g for 30 min, the solute was resuspended in complete UltraCulture (in the same volume as the loaded sample), while the eluate was reloaded onto a column with a 3 kDa membrane cut-off and centrifuged at 2700×g for 35 min. Finally, the eluate was harvested and the solute was resuspended in complete UltraCulture medium. To analyze the potential interactions between factors, for several experiments we mixed different fractions. All the fractions were immediately tested for the ability to inhibit lymphocyte proliferation, as described below.

**Table 1:** Cytokine quantification in UltraCulture medium, CM-hAMTC and CM-hAM.

	UltraCulture		CM-hAMTC		CM-hAM	
	Median	MAD	Median	MAD	Median	MAD
**TNF-a**	5	4	44	38	49	43
**Fas-l**	0	0	8	8	6	6
**G-CFS**	0	0	2271	287	2789	190
**GM-CFS**	0	0	916	602	322	14
**IL-2**	12	2	17	4	25	7
**IL-6**	0	0	>10000	ND	>10000	ND
**IL-8**	0	0	2591	162	11527	673
**IP-10**	0	0	10	9	0	0
**MIP-1a**	0	0	12583	10006	7368	1095
**MIP-1b**	3	1	3838	1519	5493	898
**OSM**	0	0	190	174	70	37
**RANTES**	0	0	265	152	53	3
**TGF-b** *	0	0	464	204	209	209
**IL-10** *	4	4	285	155	9	9

MAD: Median Absolute Deviation.

Values are expressed in pg/ml.

* quantified by ELISA.

### Lymphocyte Proliferation Test

Lymphocyte proliferation was induced either by stimulating PBMC (1×10^5^/well in 96-well-plate) by the addition of anti-CD3 (Orthoclone OKT3, Janssen-Cilag), (final concentration of 12.5 ng/ml) or by the co-culture with irradiated allogeneic “stimulator” PBMC [mixed leukocyte reaction (MLR)] at a 1∶1 ratio. All cultures were carried out in triplicate in complete UltraCulture medium, and lymphocyte proliferation was assessed by adding [^3^H]-thymidine for 16–18 hours after 3 (for PBMC + anti-CD3 stimulation) or 5 (for MLR) days of culture.

Proliferation inhibitors tested in these experiments were: (*a*) irradiated (3000 cGy) hAMTC or BM-MSC (1×10^5^/well) put in contact with PBMC, in the absence or presence of 0.5 mM DL-methyl-tryptophan; (*b*) different amounts (50, 75, 100 μl/well) of CM-hAMTC, CM-hAM or CM-BM-MSC, with or without the addition of FBS (5%) and essential/non-essential amino acids (Sigma); (*c*) different amounts of CM-hAMTC and CM-hAM obtained after temperature- or proteinase K- treatment or after column fractionation; (*d*) different amounts of CMs generated after culture of hAMTC or hAM in presence of specific inhibitors; (*e)* 100 μl of different CMs after the addition of indomethacin (10 μM), DL-methyl-tryptophan (0.5 mM), L-NAME (1 mM), a mixture of PGs (mix 1: PGE2 500 ng/ml + PGD2 100 ng/ml + PGF2α 25 ng/ml or mix 2: PGE2 25 ng/ml + PGD2 6 ng/ml + PGF2α 10 ng/ml, all purchased from Cayman Chemical) or neutralizing antibodies against IL-10 (1 μg/ml, BD), IL-6 (1 μg/ml, BD), HGF (1 μg/ml, R&D Systems) and TGF-β (1 μg/ml, R&D Systems); (*f*) different amounts (from 10 pg/ml to 5 μg/ml) of PGE2, PGD2, PGI2, PGF2α or a mixture of these PGs (mix 1: PGE2 500 ng/ml + PGD2 100 ng/ml + PGF2α 25 ng/ml; mix 2: PGE2 25 ng/ml + PGD2 6 ng/ml + PGF2α 10 ng/ml).

### NO Quantification

NO production in CM-hAMTC and CM-hAM was measured as nitrite (NO_2−_) accumulation, using the Griess reagent (G4410-Sigma Aldrich) and following manufacturer’s instructions. Serial NaNO_2_ dilutions in water were used to produce a standard curve. Absorbance was measured at 540 nm with a microplate reader (DV990/BV6-GDV).

### Prostanoid Quantification

Total prostanoids, prostaglandin (PG) E2, PGD2 and PGF2α quantification in CM-hAMTC and CM-hAM were obtained using the *Cox Inhibitor Screening Assay Kit, Prostaglandin E2 EIA kit, Prostaglandin D2 EIA kit* and *Prostaglandin F2α EIA kit* respectively (all purchased from Cayman Chemical) according to the manufacturer’s instructions. Absorbance was measured at 405 nm with a microplate reader. Since the manufacturer specifies that PGD2 monoclonal antibodies possess 92% cross reactivity with PGF2α, the amount of PGD2 was calculated by subtracting 92% of the value obtained for PGF2α from the value obtained with the PGD2 antibody.

### Cytokine Assay

The levels of different cytokines in CM-hAMTC and CM-hAM were determined using a multiple cytometric beads array system (CBA Flex set, BD Biosciences), according to the manufacturer’s instructions. The following cytokines were measured: IL-2, IL-6, IL-8, tumor necrosis factor (TNF)-α, Fas-l, granulocyte colony-stimulating factor (G-CSF), granulocyte-macrophage colony-stimulating factor (GM-CFS), interferon-inducible protein (IP)-10, monocyte chemoattractant protein (MIP)-1α and β, oncostatin M (OSM) and RANTES (regulated upon activation, normal T-cell expressed and secreted). Samples were acquired with a FACSAria and analyzed with FCAP Array software (BD Biosciences).

The levels of TGF-β and IL-10 were measured by sandwich enzyme-linked-immunosorbent assay (ELISA) using a commercially available coupled antibody (respectively 555052–555053, and 554499–554497 from BD Biosciences) according to the manufacturer’s instructions. Concentrations were calculated from standards curves, and absorbance was measured at 405 nm with a microplate reader.

### Kynurenine/tryptophan Quantification

For these experiments the following were tested: CM-hAMTC, CM-hAM and CM derived from control MLR (CM-MLR) or from MLR performed in the presence of hAMTC (CM-MLR-hAMTC), with or without the addition of 0.5 mM DL-methyl-tryptophan (CM-MLR-hAMTC+MT), which were harvested after 5 days of culture.

One ml of phosphate buffer at pH 6.5 and 2 ml trichloroacetic-acid were added to 100 μl of CM samples. The different CMs were incubated for 30 min at 50°C and then centrifuged for 15 min at 4000 rpm. Two ml of the supernatant were then used for solid phase extraction (SPE) using OASIS® HLB cartridges 1cc. Elution was ensued with 90% acetonitrile. The eluate was then dried by nitrogen and re-dissolved in 200 µl mobile phase for HPLC analysis. IDO activity was measured as the ratio between kynurenine and tryptophan concentration determined by HPLC.

### Statistical Analysis

Data are expressed as mean ± SD. Student’s *t*-test were use to assess differences between groups. Raw P-values were adjusted by Holm-Bonferroni’s procedure for multiple comparison and differences were considered statistically significant for *p* values <0.05.

**Table 2:** Prostanoids quantification in UltraCulture medium and CM derived from untreated or indomethacin treated hAMTC and hAM.

	UltraCulture	CM-hMATC		CM-hAM	
	NT	NT	Indomethacin treated	NT	Indomethacin treated
**Total prostanoids**	<1	542±280	5.8±4	69±6	16±5
**PGE2**	<1	390±146	<1	24±2	<1
**PGD2**	<1	67±1	<1	6±1	<1
**PGF2a**	<1	27±13	<1	10±1	<1

Values are expressed in ng/ml.

## Results

### CM-hAMTC and CM-hAM Inhibit Lymphocyte Proliferation

We have previously demonstrated that freshly isolated hAMTC inhibit lymphocyte proliferation *in vitro*, both when cultured in contact and in transwell settings [32]. This suggests that the anti-proliferative effect of hAMTC is associated with the production of yet unknown soluble factor(s). Herein we demonstrate that conditioned medium (CM) generated from the culture of freshly isolated non-stimulated hAMTC (CM-hAMTC) and from the culture of fragments of the amniotic membrane (CM-hAM), possess the ability to inhibit anti-CD3 stimulated-T lymphocyte proliferation in a dose dependent manner (Fig. 1A). We have also observed that CM derived from hAMTC plated at lower concentration present with lower inhibition to the CM derived from higher cell concentrations (Fig. 1A). Moreover we observed that both hAMTC at passage 3 and the CM derived from these cells (CM-hAMTC p3) maintain their anti-proliferative effects (Fig. 1B). In contrast, even if BM-MSC after 3 culture passages were also able to inhibit T cell proliferation, CM derived from the culture of these cells was not able to reduce T cell proliferation (Fig. 1B), in accordance to previously reported findings [38], [39].

Furthermore, the addition of essential and non-essential amino acids and FBS (5%) to the different CMs generated from hAMTC did not change their inhibitory ability (data not shown).

### Neutralizing Antibodies Against IL-10, IL-6, HGF and TGF-β are not Able to Significantly Revert the CM’s Inhibitory Effect

We analyzed a panel of inflammation-related molecules and their quantifications are shown in Table 1. Both CM-hAMTC and CM-hAM contain TNF-α, OSM, RANTES, GM-CFS, G-CFS, IL-6, IL-8, MIP-1α, MIP-1β, and TGF-β, but not Fas-ligand, IL-2 and IP-10. In contrast to CM-hAMTC, CM-hAM does not contain IL-10.

To evaluate the involvement of several cytokines known to elicit anti-proliferative effects in MSC from other sources (such as IL-10, IL-6, HGF, TGF-β) [13], we performed lymphocyte proliferation tests with CM-hAMTC and CM-hAM in the presence of neutralizing antibodies against IL-10, IL-6, HGF and TGF-β.

The neutralization assay revealed that HGF and TGF-β are not involved in the CM inhibitory mechanism (Fig. 2B–C). Meanwhile, the addition of neutralizing antibodies against IL-6 and IL-10 showed a minimal reversion effect without reaching a statistical difference (Fig. 2, panel A and D). Similar minimal reversion was seen also when we used a cocktail of neutralizing antibodies against IL-10, IL-6, HGF and TGF-β (Fig. 2E).

### Temperature Changes do not Influence the Anti-proliferative Effect of CM-hAMTC and CM-hAM

To investigate whether the inhibitory potential of CM-hAMTC and CM-hAM is temperature-sensitive, we studied the anti-proliferative action of these CMs after their exposure to temperature changes. After three frost-defrost cycles, CM-hAMTC and CM-hAM showed the same inhibitory effect as the corresponding untreated CMs (Fig. 3A). CMs treated for 30 min at 60°C or 80°C still retained their anti-proliferative effect (Fig. 3B). The inhibitory potential was also preserved when these CMs were stored at −80°C for more than 1 year (data not shown).

### Proteinase K Treatment does not Affect the Anti-proliferative Activity of CM-hAMTC and CM-hAM

To determine whether the inhibitory component(s) in CM-hAMTC and CM-hAM has a proteinaceous feature, we developed a protocol to remove protein molecules from CMs based on proteinase K treatment. In our experimental condition six hours of proteinase K treatment caused the digestion of more than 90% of proteins present in the two CMs (Fig. 4A). Despite protein depletion, CM-hAMTC and CM-hAM maintained their anti-proliferative activity (Fig. 4B).

### Molecular Weight of the Inhibitory Factor(s)

To establish the potential molecular weight of soluble inhibitory factor(s) present in the CM-hAMTC we subjected the CM to column fractionation. All the obtained fractions were then tested for their effects on lymphocyte proliferation.

The fraction containing molecules with a molecular weight <3 kDa (Fig. 5A, grey bars) was the only fraction maintaining an inhibitory effect. Meanwhile, fractions obtained by unconditioned medium and used as controls did not show any inhibitory effect (Fig. 5B). The results represented are obtained using 100 μl of CM, however the same trend was observed using 50 or 75 μl of CM (data not shown).

The level of inhibition achieved with the fraction containing molecules <3 kDa was significantly reduced with respect to the level of the unfractionated CM (Fig. 5A, white bar).

In order to exclude the loss of components during fractionation protocol, all the fractions were re-pooled together and tested (Fig. 5A, CM-hAMTC fraction mix, white-grey-striped bar). CM-hAMTC fraction mix showed the same anti-proliferative effect of the unfractionated CM (Fig. 5A, white bar).

Then, to find out whether soluble factor(s) present in different fractions may cooperate to exert an inhibitory ability, we mixed the single fractions two by two and tested the effects of these new preparations on T cell proliferation. All the mixes containing the fraction <3 kDa had an inhibitory effect (Fig. 5A, black-grey-striped bars), whilst only the mix containing the fraction of molecules >100 kDa and <3 kDa exerted an inhibitory effect statistically higher than that of the fraction <3 kDa alone. Furthermore, this mix had an inhibitory ability comparable to that of the unfractionated CM. The preparation obtained by mixing the fraction 100–30 kDa and the fraction <3 kDa also had an inhibitory effect higher than the fraction <3 kDa alone, but this difference did not reach a statistical significance.

### Prostaglandins but not NO or IDO are Involved in CM-hAMTC and CM-hAM Inhibitory Effect

Based on the results obtained from the studies of the molecular weight and the indication of the non-proteinaceous nature of the unknown inhibitory molecule(s), our next goal was to unravel the identity of these molecule(s). In order to do so, we evaluated the involvement of known actors in immunomodulation that have low molecular-weight and are not proteins, such as NO, kynurenine (a tryptophan metabolite) and PGs, in the anti-proliferative ability of CM-hAMTC and CM-hAM.

During CM production we therefore specifically inhibited: *i)* the NO synthesis, using L-NAME (L-Nitro-Arginine Methyl Ester), which is an inhibitor of NO synthase; *ii)* the activity of IDO, which is one of the key enzymes of kynurenine biosynthesis [40], using DL-methyl-tryptophan and *iii)* the PGs production, using cyclooxygenase (COX) inhibitors (such as indomethacin, ketoprofen and niflumic acid). CMs obtained after inhibition of these factors were then tested for their effect on activated T-lymphocyte proliferation.

First, we observed that both CMs contain a low amounts of NO, basically the same concentration as that found in the control medium (Fig. 6A). In addition, CM derived from hAMTC or hAM cultured in presence of 1 mM L-NAME had similar inhibitory effect of CMs derived from untreated samples (Fig. 6B). Taken together, these data indicate that NO is not responsible for the inhibitory effect of CM-hAMTC and CM-hAM, neither directly nor as mediator for the production of other inhibitory factors.

The measurement of IDO activity in CM-hAMTC (determined as the ratio kynurenine/tryptophan) revealed that hAMTC possess IDO activity when cultured alone, which increases when the cells are co-cultured with a MLR (as measured in the CM-hAMTC-MLR) (Fig. 6C). Despite the fact that the addition of DL-methyl-tryptophan during co-culture experiments dramatically decreases the IDO activity as measured in the CM (CM-MLR-hAMTC+MT) (Fig. 6C), the inhibitory effect of hAMTC on lymphocyte proliferation did not decrease in presence of DL-methyl-tryptophan (Fig. 6D).

To further support the finding that the inhibitory effect of CM-hAMTC and CM-hAM seems independent of IDO activity, we added DL-methyl-tryptophan during preparation of CMs (*i.e.* during hAMTC and hAM cultures). These CMs showed comparable inhibitory ability with respect to the CMs derived from untreated samples (Fig. 6E).

Quantification of prostanoids in CM-hAMTC and CM-hAM revealed that amniotic cells produce a high amount of COX products that could be partially responsible for the anti-proliferative effect of CM (Table 2). Indeed, CM-hAMTC and CM-hAM contain PGE2 (390±146 ng/ml and 24±2 ng/ml, respectively), PGD2 (67±1 ng/ml and 6±1 ng/ml, respectively), and PGF2α (27±13 ng/ml and 10±1 ng/ml, respectively). The involvement of PGs in the CM’s inhibitory effect was confirmed by the addition of COX-inhibitor during hAMTC or hAM culture. The indomethacin treatment decreased the concentration of all prostanoids in both CM-hAMTC and CM-hAM, as shown in Table 2, and interestingly, the anti-proliferative ability of these CMs were partially reverted (Fig. 6F). Similar results were also obtained using other COX inhibitors, such as ketoprofen and niflumic acid (Fig. 6G–H). Even treatment with three COX inhibitors together (indomethacin, ketoprofen and niflumic acid) during CM-hAMTC and CM-hAM production did not lead to a complete reversal of inhibitory effect (Fig. 6I).

In order to determine if PGs are partially or entirely responsible for the CMs’ inhibitory effect, we added exogenous PGs to anti-CD3 stimulated T cells. While the addition of PGI2 did not inhibit T cell proliferation, the addition of PGF2α, PGD2 and PGE2 had a slight inhibitory effect which was detectable when used at concentrations of 10000 ng/ml, 1000 ng/ml and 5 ng/ml, respectively. Thus, PGF2α and PGD2 display an inhibitory effect when used at a higher concentration than that we observed in CMs (Fig. 7A and Table 2), suggesting the lack of involvement of these in CM inhibitory effect. Meanwhile, PGE2 induces a slight reduction of T cell proliferation also when used at concentration comparable to that found in CM-hAMTC (390±146 ng/ml) and CM-hAM (24±2 ng/ml) (Fig. 7A and Table 2). Interestingly, the addition of PGE2 induced a consistent and unchanging inhibitory effect on cell proliferation in the range between 1000 ng/ml and 25 ng/ml (Fig. 7A).

With the aim to mimic the composition of PGs present in CM-hAMTC or in CM-hAM, and to evaluate if there could be some cooperation between the different PGs, we sought out to determine the effects that a mix of PGs has on T cell proliferation. As shown in figure 7B–C the addition of a mix of PGs (PGE2 500 ng/ml + PGD2 100 ng/ml + PGF2α 25 ng/ml or PGE2 25 ng/ml + PGD2 6 ng/ml + PGF2α 10 ng/ml) on stimulated PBMC inhibited the cell proliferation, but clearly at lower than that observed with CM-hAMTC and CM-hAM.

To further address the role of PGs in the CMs’ inhibitory effect, we also added PGs to anti-CD3 stimulated T-cells in the presence of CMs prepared following COX inhibition. We observed that the addition of a mix of PGs restored the inhibitory effect of the COX-inhibited CMs to approximately the level observed with CM from untreated sample (Fig. 7B–C). This suggests that some other factor(s) present in the CMs could partecipate with PGs to the CMs’ inhibitory effects.

### CM-hAMTC and CM–hAM do not Induce PGs, NO and Kynurenine Production in Leukocytes during Inhibitory Effect

After having studied which molecules are directly released by hAMTC or hAM and associated with inhibitory effect, we questioned whether the CM inhibitory effect could be due to the secretion of these immunomodulatory molecules from PBMC. We therefore added CM-hAMTC or CM-hAM on PBMC stimulated with anti-CD3 in the presence of the inhibitors indomethacin, L-NAME or DL-methyl-tryptophan. The addition of indomethacin did not shown any difference in the proliferation level (Fig. 8A), suggesting that PBMC are not induced to produce PGs in presence of CMs. Similar results were obtained after the addition of L-NAME and DL-methyl-tryptophan (Fig. 8B–C), suggesting the lack of involvement of IDO and NO in immunomodulation of the CMs.

## Discussion

In this study we have demonstrated that soluble factors released in CM from the cultures of hAMTC and hAM possess the ability to inhibit T lymphocyte proliferation. Importantly, hAMTC and hAM were not stimulated prior to CM collection. This is in contrast to MSC derived from BM [38], [39], which possess an anti-proliferative ability only when cultured in the presence of activating stimuli, such as IL-1β, TNF-α or IFN-γ [14]. This underlines the functional differences when using MSC from different sources, most likely due to the heterogeneity of the MSC populations.

In our study, aimed at defining the inhibitory factor(s) produced by hAMTC, we began with the analysis of IL-6, IL-10, TGF-β and HGF, which are already documented to be released by and involved in the immunoregulatory activity of MSCs from other sources [15], [41]–[44]. We isolated hAMTC from over 80 donors, produced CM from each cell population/donor and used them in our analyses. Specific inhibition of selected molecules with neutralizing antibodies revealed that HGF and TGF-β do not participate in the anti-proliferative effect exerted on T lymphocytes by CM-hAMTC and CM-hAM, while neutralization of IL-6 and IL-10 showed a slight reversion which did not reach significative difference.

In order to characterize the nature of the factor(s) in question, we analyzed the thermostability, proteinaceous composition, and the molecular weight of molecules contained in the CM-hAMTC. We have demonstrated that the inhibitory factor(s) are temperature-stable, possess a small molecular weight (<3 k Da) and are most likely of a non-proteinaceous nature. We favour the non-proteinaceous nature of some of these compounds because CM treatment with protease which induced 90% protein degradation still did not result in a significant reduction in the capability of the CM to decrease T-cell proliferation. These observations brought us to focus on the potential involvement of small molecules. Some authors have reported that NO and IDO play critical roles in the suppression of T-cell proliferation when using BM-MSC [45], [46]. Our results clearly demonstrate that these factors are not involved, neither directly nor as mediators in the production of other inhibitory factors, as proven by blocking their synthesis pathways using selective inhibitors for IDO and NO synthase (*e.g.* methyl-tryptophan and L-NAME, respectively). No effects were seen either when the inhibitors were used on hAMTC during CM production, or during the incubation of CM-hAMTC with CD3-stimulated PBMC, thus implying that PBMC do not produce NO or IDO in reaction to (after coming in contact with) stimuli found in the CM of hAMTC.

Interestingly, we have shown that hAMTC and hAM release PGs in culture and that these are responsible for a significant part of the inhibitory effect. Moreover, the chemical-physical nature of PGs is in agreement with the high stability and low molecular weight of the factors we characterized in CM-hAMTC. Nevertheless, treatment with COX inhibitors was unable to completely abolish the inhibitory effect of CM-hAMTC and CM-hAM, suggesting the involvement of other factors. Consistent with this hypothesis is the fact that the addition of a mix of PGs (that mimics those present in CM) on stimulated PBMC induces an inhibition of proliferation but at a lower level than that observed with CM-hAMTC and CM-hAM. Furthermore, we have shown that the addition of a mix of PGs to anti-CD3 stimulated T cells in the presence of CM prepared following COX inhibition rescues the inhibitory effect to the level observed with CM from untreated hAMTC. Taken together, these results indicate that PGs are just one of the key effector molecules involved in the immunomodulatory activity of the amniotic membrane. The influence of other factors is also suggested by our investigations on the molecular weight of soluble factors in CM-hAMTC. Indeed, taken individually, the only fraction able to significantly decrease lymphocyte T cell proliferation was the one <3 kDa, even though not to the extent of the unfractionated CM-hAMTC, thus suggesting that other components are also responsible for the inhibitory effect. The inhibitory effect obtained with unfractionated or the pool of fractions was also observed by mixing the two fractions <3 kDa and >100 kDa, suggesting the contribution of molecules within these molecular weight ranges to the immunomodulatory effect of the CM from the amniotic-derived cells.

Some reports suggest that factors such as NO, PGs, and IDO activity are usually active in a multi-cellular environment, which includes not only MSC but also PBMC or other cell types [47], [48]. In order to distinguish which cells could produce PGs (MSC or PBMC), we used COX inhibitors both during the generation of CM from hAMTC and from hAM, and also during the culture of the generated CM (CM-hAMTC and CM-hAM) with stimulated PBMC. We observed that PGs are produced and detected in CM-hAMTC and CM-hAM, while it is not produced by PBMC stimulated by the CM.

It is noteworthy that at present we have performed our analyses only with CM derived from unstimulated cells. This is because our first aim was to understand which inhibitory factor(s) are produced by hAMTC in the absence of particular stimuli or particular culture conditions. Eventually, in follow-up studies we aimed to determine for the conditions that can change/enhance the spectrum of factors released by these cells. Actually, it has been reported that the exposure of human amniotic mesenchymal cells to inflammatory stimuli (such as to the action of IFN-γ or IL-1β), induces changes in the expression of some cellular surface markers [49], [50] and the upregulation of secretion of factors involved in inflammation (such as TGF-β [51], IDO [50], [51], IL-8 [49], IL-6 [49], serpin E1 [49], IP10 [49], sCD54 [49], MCP-1 [49] and PGs [51], [52]). Moreover, this exposure also increases the anti-proliferative ability of human amniotic mesenchymal cells when co-cultured in contact with PBMC [49], [50]. These fragmented results suggest that the state of activation of amnion-derived mesenchymal cells may also affect their capability to modulate cell proliferation. Likewise, we have previously demonstrated that hAMTC inhibit the proliferation of T lymphocytes [32] and also of cancer cell lines from hematopoietic and non-hematopoietic origin [37], in both cell-cell contact and transwell co-cultures, and with a higher level of inhibition in a cell-cell contact settings. It could be possible that the contact between cells leads to the release of some other factors involved in the inhibition of the lymphocytes proliferation. Therefore, it remains to be elucidated if there is any qualitative and/or quantitative difference in the pattern of components of the supernatants, including PGs, when obtained from stimulated cells and from cells that are cultured in cell-cell manner.

This study did not reveal any appreciable differences concerning the inhibition of lymphocyte proliferation between the CM derived from the whole amniotic membrane and the CM derived from cells isolated from the membrane. This suggests that the amniotic membrane could be an interesting source of soluble factors, without referring to extensive cell preparation. Even though the presence of the cells derived from both the mesenchymal and the epithelium layers of amniotic membrane might contribute to produce a supernatant with different composition.

In conclusion, in this study we have shown for the first time that the anti-proliferative effect of hAMTC is not related to the presence of external stimuli. hAMTC and hAM are indeed able to release soluble factors with inhibitory effects when cultured in non-stimulated conditions, proving that the effect is an intrinsic characteristic. Moreover, we have also provided evidence that this effect seems to be mediated by low molecular weight, non-protein, thermostable compound(s) present in CM-hAMTC and CM-hAM. Finally we provide evidence that prostaglandins are one of the key effector molecules in the immunomodulatory activity of the human amniotic membrane, while this activity minimally involves IL-6 and IL-10, but not IDO, NO, TGF-β or HGF instead proposed to be involved in anti-proliferative action of MSC of other sources.
